# Using Virtual Street Audits to Understand the Walkability of Older Adults’ Route Choices by Gender and Age

**DOI:** 10.3390/ijerph13111061

**Published:** 2016-10-28

**Authors:** Katherine Brookfield, Sara Tilley

**Affiliations:** University of Edinburgh, Edinburgh EH3 9DF, UK; katherine.brookfield@ed.ac.uk

**Keywords:** older adults, built environment, physical activity

## Abstract

Walking for physical activity can bring important health benefits to older adults. In this population, walking has been related to various urban design features and street characteristics. To gain new insights into the microscale environmental details that might influence seniors’ walking, details which might be more amenable to change than neighbourhood level factors, we employed a reliable streetscape audit tool, in combination with Google Street View^™^, to evaluate the ‘walkability’ of where older adults choose to walk. Analysis of the routes selected by a purposive sample of independently mobile adults aged 65 years and over living in Edinburgh, UK, revealed a preference to walk in more walkable environments, alongside a willingness to walk in less supportive settings. At times, factors commonly considered important for walking, including wayfinding and legibility, user conflict, kerb paving quality, and lighting appeared to have little impact on older adults’ decisions about where to walk. The implications for policy, practice, and the emerging technique of virtual auditing are considered.

## 1. Introduction

Walking is the most common form of physical activity amongst older adults [1] and the most common mode of transport [2]. Evidence suggests that regular walking might provide important health benefits for this population [3]. Walking more than 4 h a week may, for instance, reduce the risk of hospitalisation for cardiovascular disease events in both older men and women [3].

Evidence suggests that older adults’ walking behaviours are related to objective and perceived environmental characteristics [4]. At the neighbourhood level, mixed land uses [5], proximity to recreational uses [6], and density [7] have been associated with walking. At street level, various factors including pedestrian infrastructure [7,8], pedestrian crossings, street lighting, and traffic volume [2] have been identified as important, although not by all studies [8]. 

To gain new insights into the street-level environmental details that might encourage or discourage walking in older adults, items which might be more amenable to change than neighbourhood level factors [8], we used a desk-based audit tool to assess the ‘walkability’ of routes that older adults choose to walk within the community. 

Walkability commonly refers to the extent to which the built environment encourages walking by providing a safe and comfortable setting for pedestrians [9]. Few studies have focused on either older adults’ route choices, or the walkability of these routes, although an appreciation of the environments in which older adults elect to walk might facilitate a better understanding of the complex relationship between this group’s walking behaviours and the built environment. Rather, studies have usually attended to the measured and/or perceived physical characteristics and urban design features of neighbourhoods within which older adults live and investigated possible associations between these items and levels of walking or physical activity (see [10,11,12,13]). The few studies which have looked at where older adults choose to walk have usually concentrated on specific types of walking or subsets of the older population. These include an investigation into the relationship between the environmental characteristics of path segments and path choice in active older adults living in purpose built, campus-type retirement communities [14]; an examination of the perceived physical characteristics of routes chosen by older adults for walking for transport [15]; and the influence of observed neighbourhood characteristics on the routes older adults’ select to access specific destinations (e.g., shops, health care facilities) [16].

Amongst the few studies which have examined the walkability of older adults’ route choices, walkability has typically been measured through in-person environmental audits (see [4,14,17]). Such audits can be costly and time intensive, which can limit the size of the area audited. Safety can be a concern for the auditors, while local residents may perceive the audit as intrusive [18]. Increasingly, sophisticated online mapping services that provide 360-degree imagery of street-level scenes, such as Microsoft’s Streetside^®^ (Microsoft, Redmond, WA, USA) and Google Street View^™^ (Google^,^Inc., Mountain View, CA, USA) offer the possibility of a less costly, less intrusive, less onerous alternative to in-person audits [19,20,21]. Several studies have used such mapping services to undertake desk-based audits of an area’s environmental supportiveness for walking (see [18,19,21]) and cycling [22]. Charreire et al. [23] completed a systematic literature review on the use of these services to assess aspects of the built environment related to dietary behaviour and physical activity. Other studies have employed such mapping services to assess urban street greenery [24], the presence of smokefree signage [25] and characteristics contributing to pedestrian injuries [26]. Importantly, this desk-based approach has been identified as a reliable alternative to in-person audits [19,20,21,27]. 

Seeking to gain new insights into the street-level factors that might influence walking in seniors, while further developing the area of virtual auditing, we employed the Forty Area STudy street VIEW (FASTVIEW) audit tool (see Materials and Methods), in combination with Google Street View™, to examine the walkability of where older adults choose to walk within the community. 

Engaging with a purposive sample of independently mobile older adults aged 65 years and over, we found a preference to walk in more walkable environments alongside, on occasion, a willingness to walk in less supportive settings. We consider here the implications for policy, practice, and the emerging technique of virtual auditing. 

## 2. Materials and Methods 

We asked 19 purposively sampled older adults aged 65 years and over to select a route to walk with a researcher (see Participants). Participants provided written consent and ethical approval was provided by the University of Edinburgh through Edinburgh College of Art Research, Ethics and Knowledge Exchange Committee (ref. 09/04/2015). The Global Positioning System (GPS) tracking application Google My Tracks™ (Google Inc., Mountain View, CA, USA) (no longer available) was used to record the route, speed, and distance of each walk. Google Maps™ (Google Inc., Mountain View, CA, USA) was used to plot the routes and measure distances. Subsequently, a desk-based assessment of the walkability of each route was performed using the FASTVIEW audit tool and Google Street View™. 

### 2.1. The FASTVIEW Audit Tool

The FASTVIEW audit tool was developed by Griew and colleagues [19] from the UK-based Forty Area STudy in collaboration with a UK-based transport consultancy, Transport Research Laboratory (TRL). It builds on TRL’s Pedestrian Environment Review System which was created to facilitate an objective review of conditions for pedestrians. The FASTVIEW tool focuses on nine categories of neighbourhood characteristics—pavement width and obstructions, pavement surface quality, kerb paving quality, road permeability, way finding and legibility, lighting, personal security, user conflict, and environment quality—that have been shown to be important to walking. Each category includes up to three discrete factors, and each factor is scored against a set of ‘levels’. The levels move from positive to negative assessments. For example, for the category ‘pavement width and obstructions’, the three factors are pavement width, street furniture placement, and cars parked on pavement. The factor ‘pavement width’ is scored against the levels, moving from positive to negative assessments: >3 m; 2–3 m; <1 m; no pavement. The factor ‘street furniture’ is scored against the levels, again moving from positive to negative: aligned to the side; poorly placed; N/A (no pavement present). Finally, the factor ‘cars parked on pavement’ is scored against the levels: no cars on pavement; cars on pavement; N/A (no pavement present). Full details of the tool are provided in Griew et al. [19]. When applied to a street section, an overall score for each category is determined by combining the scores achieved for each factor within that category. Where factors are consistently scored positively, the category is rated as ‘good’. Where factors are consistently scored negatively, the category is rated as ‘poor’. If there is no consistency in the scores, then the category is rated as ‘fair’. 

The FASTVIEW tool has been found to offer a reliable measure of street characteristics commonly associated with walking [19]. Assessed using Cohens’ kappa statistic, Griew et al. [19] found that intra-rater reliability results were high for the tool’s nine street characteristics with an average of 81% (kappa coefficient (*k*) = 0.4) agreement between results in a first and second audit. Assessed using Fleiss’ kappa statistic, they found that inter-rater reliability results were high for most of the street characteristics with an average of 71.7% (*k* = 0.3) agreement, although, for three categories—pavement quality (51.3%, *k* = −0.009), road permeability (58.3%, *k* = 0.143) and street lighting (57.7%, *k* = −0.075)—reliability results were low [19]. Lastly, assessed using Cohens’ kappa statistic, they found that criterion reliability (i.e., the degree of agreement between in-person and desk-based audits) results were high across all street characteristics with an average of 84% (*k* = 0.7) agreement [19]. This degree of reliability helped prompt the tool’s use. Additionally, the research offered an opportunity to consider the tool’s effectiveness in a large, complex urban setting—Edinburgh, the capital city of Scotland—a densely populated city of 493,000 people [28] and an United Nations Educational, Scientific and Cultural Organization (UNESCO) World Heritage site.

### 2.2. Participants

Purposive sampling was used to recruit 19 older adults from Edinburgh, UK. Participants were recruited using a variety of methods including: an advert placed in a local newspaper, leaflet distribution, and via local community and older people’s organisations and networks. This resulted in 22 responses. Three women subsequently declined to be interviewed due to time restrictions. Participants (mean age = 74.9, standard deviation = 7.9, age range 65–91 years) had to be able to walk for at least 15 min unaided by another person and be aged 65 years or over. A diverse sample was deliberately sought to ensure a wide age range and even gender split. Residential location was also taken into account with participants recruited from diverse neighbourhoods in terms of socioeconomic profile and environmental design, located across Edinburgh. 

Table 1 in the Results section provides an overview of the sample and a summary of the selected routes. The 19 participants each selected a route to walk with a researcher. Participants were asked to undertake a ‘typical walking journey’, defined by them, which they would usually make in the course of a week. Due to health and safety concerns, and ethical considerations, participants were asked to select a short walk that would take around 20–30 min to complete. This direction notwithstanding, wishing to understand the routes that older adults chose for themselves, no further guidance on route choice was provided. Participants were not, for instance, instructed to only select routes located within their residential neighbourhood (howsoever defined). 

### 2.3. Analysis

Following the approach adopted by Griew et al. [19], the 19 routes were divided into road sections termed ‘links’. As far as possible, these links stretched between road junctions. The minimum link length was set at 50 m and the maximum at 300 m [19]. Each link was audited using the FASTVIEW tool and rated as ‘good’, ‘fair’, or ‘poor’ in each of the nine neighbourhood characteristic categories based on the combined scores from the factors associated with these categories. Each route was rated as ‘good’, ‘fair’, or ‘poor’ in each of the nine categories based on the combined scores of the route’s composite links. Where most links (around 75% and over) were rated as ‘good’ in a given category, the route as a whole was rated as ‘good’ in that category. Similarly, where most links were rated as ‘poor’ in a category the route as a whole was rated as ‘poor’ in that category. Where there was no consistency in the ratings, the route was rated as ‘fair’ in that category. Approximately 26 km of route, contained within 147 links, were audited in the study. The average link length was 179 m.

## 3. Results

The 19 routes (summarised in Table 1) ranged from 1 km to almost 5 km, the median length being 2.5 km. Fifteen routes supported walking for recreation and four routes walking for transport, or for another specific purpose—walking to exercise a pet in the case of our participants. Nine were circular, six were linear consisting of an outward leg, and four were linear consisting of an outward and return leg. Routes took in a variety of environmental settings including quiet residential streets, busy shopping districts, historic sites, the city centre and neighbourhoods on the edge of the city centre. Fourteen routes included interactions with green space and seven interactions with blue space (two included interactions with both types of space). The term ‘blue space’ refers to aquatic environments, or environments with aquatic elements such as lakes, rivers, streams, and coastlines [29]. The median walk time was 66 min. In our view, several factors explained this longer than anticipated walk time. Our participants appeared to be physically active with many claiming to be regular walkers. This existing interest and participation in walking, attachments to particular routes, a wish to share these routes with others, good weather (we completed the walks in summer), and, for a few, the opportunity for company, to stroll and chat with a researcher, explained the long walk times.

Table 1 disaggregates the routes by gender and age. Routes selected by women (*n* = 10) tended to be shorter than those selected by men (*n* = 9), they always supported walking for recreation, they were more likely to be circular and they were less likely to incorporate busy city centre environments. Men and women were similarly likely to select routes that incorporated interactions with green space, although women’s routes were more likely than men’s to interact with blue space. 

Routes selected by adults aged 75 years and over (*n* = 8), sometimes termed the ‘old, old’ [30], were fractionally shorter than those selected by adults aged 65 to 74 (*n* = 11) and were more likely to support walking for recreation. The routes selected by these two age groups were similarly likely to include interactions with green space and blue space. In those selected by the 75 and over age group, the first interaction with this space occurred much later. 

Table 2 provides an overview of the route walkability scores. Route walkability, as determined through application of the FASTVIEW audit tool, was generally good across all 19 routes although occasionally, and on certain measures—wayfinding and legibility, user conflict, kerb paving quality, and lighting—it was poor.

All routes included sections which could not be audited using the FASTVIEW tool. These route sections took in footpaths and trails through public parks, cemeteries, and car parks, along local railway lines, riverbanks, canals, and beaches. On average, approximately 49% of a route was located within such ‘non-street’ environments. The FASTVIEW tool’s focus on streets, and its reliance on Google Street View™, made it inappropriate and impractical to assess these ‘non-street’ settings. 

Table 3 disaggregates the walkability scores by gender. Women tended to select routes that scored well on pavement width and obstructions, pavement surface quality, personal security, and environment quality. Men also often selected routes that scored well on environment quality and personal security. Footpaths, trails, and other ‘non-street’ environments constituted a greater proportion of the routes selected by men than they did the routes selected by women. Women were more likely than were men to select routes that achieved a poor score in one of the FASTVIEW tool’s nine categories.

Table 4 compares the walkability scores by age group. Participants aged 75 years and over tended to select routes that scored well on environment quality, pavement surface quality, personal security, and user conflict. Participants aged between 65 and 74 also tended to select routes that scored well on environment quality and personal security, plus kerb paving quality and lighting. Footpaths, trails, and other ‘non-street’ environments constituted a greater proportion of the routes selected by those aged 65 to 74 than they did the routes selected by those aged 75 and over, whereas the routes selected by this latter age group were more likely to achieve a poor score in one of the FASTVIEW tool’s nine categories.

## 4. Discussion

Route walkability, as determined through the application of the FASTVIEW audit tool, was generally good across all 19 routes although occasionally, and on certain measures, wayfinding and legibility, user conflict, kerb paving quality, and lighting, it could be poor. This may suggest that older adults prefer to walk in more walkable environments, as determined by the FASTVIEW tool, but are willing to walk in less supportive settings. Further, it suggests that certain pro-walking factors, contrary to findings reported elsewhere, e.g., Day [31] on kerb paving quality, might have little bearing on older adults’ decisions about where to walk. Pertinent here, a systematic review of studies into the relationship between the environment and physical activity in older adults found that most of the investigated relationships were non-significant [4]. This might suggest that the environment has little impact on walking and other forms of physical activity in this age group. The authors of the review stress, however, that this is not necessarily the case arguing that the failure to find significant relationships might ‘reflect some methodological issues within this developing research field’ [4]. For example, they suggest that the tendency for studies to focus on total physical activity might obscure relationships between the environment and specific types of physical activity [4]. More studies, especially prospective studies, into possible links between the environment, walking, and other varieties of physical activity in this group are needed to draw firm conclusions on this matter [4]. 

The walkability of the 19 routes was determined in large part by the walkability of Edinburgh. Presenting urban design features and neighbourhood characteristics commonly associated with walking, and considered by the FASTVIEW tool, including good pedestrian infrastructure, greenery, and aesthetically pleasing/high quality environments [15,32], Edinburgh appears highly walkable. Consequently, the degree to which a route’s walkability ‘score’ was a function of a participant consciously selecting a path that performed well on pro-walking environmental factors, and the degree to which it was a function of the local environment performing well on those factors, is debateable.

Connecting to studies which find differences between men and women [15,33,34], and younger and older age groups [35], in the environmental factors that might matter in walking, there were gender differences, and to a lesser extent age differences, in route setting and walkability, plus route structure, length, and function. Women were more likely to select routes that performed poorly against measures commonly associated with walking, particularly wayfinding and legibility. Pertinent here, some evidence suggests that men and women may employ different wayfinding strategies with men relying more on environmental reference points and women more on route knowledge [36]. Observed by this study, greenspace was particularly important for men in decisions about where to walk. Larger proportions of the routes selected by men included or were located within greenspace. Michael et al. [37] found that proximity to a park or trail was associated with maintaining or increasing time spent walking in older men (although the association was only found in men living in neighbourhoods with a high socioeconomic status). The subtle differences in route choices identified between the two age groups is likely to be a function of the seemingly similar levels of physical activity found across the sample, with many participants claiming to be regular walkers. Due in part to the inclusion/exclusion criteria employed within the study, which required individuals to be able to walk for at least 15 min unaided by another person, those aged 75 years and over were observed to be no frailer, nor any less active, than those aged 65 to 74 years; however, no formal assessments of physical function or physical activity were performed.

Footpaths, trails, and other ‘non-street’ environments were a key feature of many of the 19 routes. Although further research is needed, this suggests that for older adults, often ‘green’ and ‘blue’, non-street settings are preferred over streets and roads as favoured environments in which to walk. This links to studies which have found associations between walking, physical activity and access to green space in older adults [32,37,38,39], although others have found that this age group is averse to isolated trails through wooded areas [32] and can avoid areas of green space when walking in urban districts [16]. 

The presented findings could have implications for policy and practice. To encourage walking amongst older adults, planners, designers, and others could attend to the design, provision, and quality of microscale environmental details, such as pavement quality, which have been associated with walking in this population [15] and which seemed to matter to our participants. Some practitioners and organisations are already doing this. In the UK, the ongoing development of London’s Queen Elizabeth Olympic Park, host of the 2012 Olympic Games, is informed by a set of Inclusive Design Standards [40] and an Inclusive Design Strategy [41], which provide guidance on such items as walking surfaces, lighting, and signage. In America, various communities, regions, and states have adopted ‘Complete Streets’ policies which, in seeking to create streets that accommodate all types of users, attend to such details as the provision and quality of pavements [42]. To promote walking in older adults, health and aligned professionals could develop and situate walking programmes targeted at this age group in green and blue spaces, given their apparent appeal to this population. Again, some practitioners are already doing this. In Edinburgh, the ‘Ageing Well’ initiative operates a series of walking groups across the city for adults aged 50 years and over, several of which undertake walks through green space and/or blue space [43]. In Hendersonville, North Carolina, USA, the older adult focused pedestrian safety programme ‘Walk Wise, Drive Smart’ mapped a series of walking routes for seniors, several of which encompassed areas of green space [44]. In Sydney, Australia, the ‘After Dinner Walking Group’ targeted at active adults aged 55 years and over completes night time walks around Sydney’s cultural highlights including, at times, its public parks [45]. 

The FASTVIEW desk-based audit tool, used in conjunction with Google Street View™, quickly and easily provided insights into various factors commonly associated with walking for the routes included within our study. Approximately 26 km of route were audited, and a route took at most around 2 h to assess. Three hundred and sixty-degree imagery of street-level scenes was available for all the street sections included within the routes. The images were high quality and recent, typically from the last two years. This supported a straightforward auditing process. Some challenges did arise, however, when streets were lined with tightly packed parked cars. Achieving a clear view of the pavement proved difficult in these situations. 

Researchers have sought to adapt environmental audits and survey tools to the attributes of a given area and/or the needs of discrete populations. The popular Neighbourhood Environment Walkability Scale (NEWS) has, for example, been modified for use with older women in the United States [46] and Chinese seniors in Hong Kong [47]. We suggest that, in certain contexts, the FASTVIEW tool might benefit from some such sensitisation. In quiet, outlying suburban areas it might be prudent to remove from the tool the items ‘traffic calming measures’ (included in the category ‘personal security’) and ‘closed-circuit television (CCTV) surveillance’ (included in the category ‘user conflict’). In such areas, these items are rare, indeed their presence might suggest problems with cut-through traffic or anti-social behaviour. However, as the tool is presently structured, their absence could lower an area’s walkability score. Further, to understand an environment’s walkability for older adults, it might be advantageous to remove, or reposition as a hindrance to walking, the item ‘tactile paving’ (included in the category ‘kerb paving quality’). Some evidence suggests that such paving complicates mobility in older adults [48]. However, as the tool is presently structured, the presence of tactile paving could raise an area’s walkability score. 

Strengths of the study include its originality; as no other study has used virtual street audits to understand the walkability of older adults’ route choices. The study has built knowledge around where older adults choose to walk, the environmental factors that might relate to these decisions, and the usefulness of desk-based audits as mechanisms for appraising an environment’s walkability. 

The paper sought to present an objective assessment of the environmental characteristics of walking routes selected by older adults and, consequently, it has not considered participants’ subjective assessments of these characteristics. An environment’s perceived walkability can differ from its objectively assessed walkability [49], while research based on objective measures can identify different relationships between walking and the environment, and different pro-walking environmental factors, than can research based on subjective measures [50]. Other limitations of the study include the use of an unrepresentative sample, which makes extrapolating to the wider population of older adults difficult. Further, it is unclear how far the walkability of the selected routes was determined by older adults’ path choices vis-à-vis the walkability of the local built environment. Finally, with the audited routes mainly supporting walking for recreation, the study is less suited to commenting on route characteristics associated with walking for other purposes, such as walking for transport. Evidence suggests that different environmental factors are relevant to different walking behaviours [14,51,52]. Future research could address these issues through the use of a larger, representative sample, drawn from a range of more and less walkable environments, and by asking participants to select different types of routes (e.g., a ‘walking for recreation’ and a ‘walking for transport’ route). 

## 5. Conclusions

Virtual street audits completed on walking routes selected by 19 purposively sampled older adults using the FASTVIEW tool and Google Street View™ revealed much variation in route length, structure, function, and environmental context. However, there was notable consistency in route walkability with most performing well on environmental factors commonly associated with walking. Patterns emerged when the routes were disaggregated by gender and age. The results suggest that older adults prefer to walk in more walkable environments but, with some routes performing poorly against certain pro-walking factors, are willing to walk in less supportive settings. Further, the results suggest that certain factors commonly considered important for walking might have little impact on older adults’ decisions about where to walk. Future research could usefully investigate this issue.

## Figures and Tables

**Table 1:** Sample and routes by gender and age.

Item	Sample	Gender		Age	
		Women	Men	65 to 74 Years	75 and over
Number	19	10	9	11	8
Age (median)	72 (Quartiles 1–3: 68, 72, 79)	72	74	70	82
Gender	10 Women, 9 Men	10	9	6 Women, 5 Men	4 Women, 4 Men
Length (km) (median)	2.5 (1.97, 2.50, 3.78)	2.45	3.35	2.55	2.50
Time taken to complete route (min) (median)	66:22 (53.82, 66.63, 76.55)	63:34	66:38	65:02	67:35
Moving time (min) (median)	64:47 (51.97, 64.78, 71)	59:55	64:47	64:44	66:19
Fastest pace (min/km) (median)	08:20 (8.03, 8.33, 10.53)	08:13	09:31	09:31	08:20
Average pace (min/km) (median)	21:22 (18.18, 21.37, 27.60)	23:53	19:45	20:15	25:23
Route function	15 Recreation 4 Transport/Other specific purpose	All routes: Recreation	6 Recreation 3 Transport/Other specific purpose	8 Recreation 3 Transport/Other specific purpose	7 Recreation 1 Transport/Other specific purpose
Route structure	9 Circular, 6 Linear (out), 4 Linear (out and return)	6 Circular 2 Linear (out) 2 Linear (out and return)	3 Circular 4 Linear (out) 2 Linear (out and return)	4 Circular 6 Linear (out) 1 Linear (out and return)	5 Circular 3 Linear (out and return)
Route context	Quiet residential streets, busy shopping districts, including city centre streets, historic areas and edge of city centre streets, greenspace, blue space	Quiet residential streets, busy shopping districts—not usually within the city centre (1 route), historic areas and edge of city centre streets, greenspace, blue space	Quiet residential streets, busy shopping districts—including city centre streets (5 routes), historic areas and edge of city centre streets, greenspace, blue space	Quiet residential streets, busy shopping districts—including city centre streets (6 routes), historic areas and edge of city centre streets, greenspace, blue space	Quiet residential streets, busy shopping districts—not city centre streets, historic areas and edge of city centre streets, greenspace, blue space
Greenspace (No. routes)	14	7	7	8	6
Blue space (No. routes)	7	5	2	4	3
Walk start point	13 Participant’s home 4 Park 1 Country road 1 University of Edinburgh	8 Participant’s home 1 Park 1 Country road	5 Participant’s home 3 Park 1 University of Edinburgh	5 Participant’s home 4 Park or area of greenspace 1 University of Edinburgh	All: Participant’s home
Distance to first interaction with greenspace/blue space (m) (median)	260 (0, 260, 500)	256	260	60	342

**Table 2:** Route walkability scores.

FASTVIEW Categories	No. (and %) Routes Rated Good	No. (and %) Routes Rated Fair	No. (and %) Routes Rated Poor	No. (and %) Routes Where Category Was N/A
Pavement width and obstructions	12 (63%)	7 (37%)		
Pavement surface quality	13 (68%)	6 (32%)		
Kerb paving quality	9 (47%)	8 (42%)	1 (5%)	1 (no kerbs) (5%)
Road permeability	6 (32%)	13 (68%)		
Way finding and legibility	10 (53%)	6 (32%)	3 (16%)	
Lighting	12 (63%)	6 (32%)	1 (5%)	
Personal security	15 (79%)	4 (21%)		
User conflict	12 (63%)	5 (26%)	2 (11%)	
Environment quality	19 (100%)			
Median % of route unable to audit as situated in ‘non-street’ environments	49% (Quartiles 1–3: 17%, 49%, 75%)			

Percentage values may not always sum to 100% due to rounding.

**Table 3:** Route walkability scores by gender.

FASTVIEW Categories	Women				Men			
	No. Routes Rated Good	No. Routes Rated Fair	No. Routes Rated Poor	No. Routes Where Category Was N/A	No. Routes Rated Good	No. Routes Rated Fair	No. Routes Rated Poor	No. Routes Where Category Was N/A
Pavement width and obstructions	8	2			4	5		
Pavement surface quality	8	2			5	4		
Kerb paving quality	3	5	1	1	6	3		
Road permeability	4	6			2	7		
Way finding and legibility	4	3	3		6	3		
Lighting	6	3	1		6	3		
Personal security	8	2			7	2		
User conflict	6	3	1		6	2	1	
Environment quality	10	0			9			
Median % of route unable to audit as situated in ‘non-street’ environments	48%				69%			

**Table 4:** Route walkability scores by age.

FASTVIEW Categories	Aged 65 to 74				Aged 75 and over			
	No. Routes Rated Good	No. Routes Rated Fair	No. Routes Rated Poor	No. Routes Where Category Was N/A	No. Routes Rated Good	No. Routes Rated Fair	No. Routes Rated Poor	No. Routes Where Category Was N/A
Pavement width and obstructions	7	4			5	3		
Pavement surface quality	7	4			6	2		
Kerb paving quality	8	2	1		1	6		1
Road permeability	3	8			3	5		
Way finding and legibility	6	5			4	1	3	
Lighting	8	3			4	3	1	
Personal security	9	2			6	2		
User conflict	6	4	1		6	1	1	
Environment quality	11				8			
Median % of route unable to audit as situated in ‘non-street’ environments	70%				47%			
